# Disease recurrence in patients with Crohn’s disease after biologic therapy or surgery: a meta-analysis

**DOI:** 10.1007/s00384-022-04254-z

**Published:** 2022-09-23

**Authors:** Sarah Kneißl, Johannes Stallhofer, Peter Schlattmann, Andreas Stallmach

**Affiliations:** 1 Department of Internal Medicine IV (Gastroenterology, Hepatology, and Infectious Diseases), Jena University Hospital, Am Klinikum 1, 07747 Jena, Germany; 2 Institute for Medical Statistics, Informatics and Data Science, University Hospital Jena, Bachstr. 18, 07743 Jena, Germany

**Keywords:** Crohn’s disease, Infliximab, Adalimumab, Resection, Recurrence

## Abstract

**Background:**

Relapse is a problem in patients with Crohn’s disease (CD) after medical therapy (including biologics) and after surgery to treat acute inflammation. It is unclear whether the recurrence rate over time is higher after surgical therapy than after continuous drug treatment.

**Aim:**

We sought to compare clinical relapse rates and the need for re-interventions (resection or therapeutic endoscopic intervention) in patients with CD.

**Methods:**

A meta-analysis was performed according to PRISMA guidelines.

**Results:**

The need for re-intervention with medication or surgery due to surgical or clinical recurrence increased over time. The recurrence rates in patients after ileocecal resection were lower than the rates under biologic therapy. The odds ratio for clinical recurrence under biologics versus after surgical treatment was 2.50 (95% confidence interval [*CI*] 1.53–4.08, *p*-value < 0.001). The odds ratio for surgical recurrence under biologics versus after surgery was 3.60 (95% *CI* 1.06–12.3, *p*-value 0.041).

**Conclusion:**

These findings support surgical resection as a treatment option in patients with CD with limited disease.

**Supplementary Information:**

The online version contains supplementary material available at 10.1007/s00384-022-04254-z.

## Introduction

Crohn’s disease (CD) is a chronic inflammatory bowel disease of unclear aetiology that usually affects young adults and children, with a peak incidence between the ages of 15 and 35. In western industrialised countries, the incidence is 6–15 per 100,000, and the prevalence is 50–200 per 100,000. In recent decades, the incidence and prevalence have increased globally [1]. Maintaining remission of CD is a central goal in clinical practice. Recurrence of CD has a strong negative impact on health and quality of life [2]. Therapeutic concepts for maintaining remission are usually based on medical therapy alone. The standard step-up concept using immunosuppressants and biologics is guided by German and international guidelines [3]. Immunomodulatory treatment aims to prevent clinical relapses and repeat surgery.

Against this background, the number of patients treated long-term with biologics such as anti-TNF antibodies has risen over the last few years. In contrast, surgery remains a treatment option. Its benefits were recently confirmed in the LIR!C trial. In that study, the biologics and surgery groups showed comparable results in terms of quality of life with no additional severe side effects [2]. However, the long-term course for patients with initial infliximab therapy was characterised by more frequent ileocecal resection. In contrast, the initially resected patients had to start infliximab therapy significantly less often due to a recurrence. These findings suggest that ileocecal resection is a viable alternative to biologics in patients with an isolated ileitis terminalis [4].

Recently, studies on the various treatment options and their effects focused on only one treatment option. Nevertheless, some patients may benefit from earlier surgery followed by biologic therapy to maintain remission. Therefore, in our meta-analysis, we included ileocecal resection, administration of biologics or a combination of both. We compared the results regarding clinical recurrence and the need for surgical re-intervention or endoscopic balloon dilatation.

The present study aimed to compare medical and surgical treatment strategies and to derive possible therapy suggestions that are likely to optimise outcomes.

## Methods

The following “Methods” section was structured according to the PRISMA guidelines.

### Eligibility criteria

Clinical trials, meta-analyses, randomised controlled trials, reviews and systematic reviews with pro- and retrospective, placebo-controlled, randomised and double-blinded designs were included. To generate the most significant number of patients, the following patients were included: non-naive patients (regarding previous medication), concomitant and post-interventional medication and previous operations. Our exclusion criteria were as follows: (1) ‘books and documents’, (2) children up to the age of twelve, (3) follow-up within 30 days and (4) mixed populations of ulcerative colitis and CD patients.

### Information sources and search strategy

We obtained full-text articles from PubMed. The date the database was last consulted was the 3rd of June 2022. To locate articles relating to outcomes after ileocecal resection for CD, our search terms included ‘Crohn's disease’, ‘ileocecal resection’, ‘recurrence’ and ‘outcome’.

For the treatment with biologics and the combination of resection and biologics, our terms included ‘recurrence of Crohn* after infliximab/adalimumab’.

Regarding the combination therapy defined by the use of biologicals after resection, the search criteria included ‘ileocecal resection and biologics Crohn*’ and ‘Crohn infliximab/adalimumab post-operative’.

### Selection and data collection process

In this meta-analysis with logistic regression, we included studies focusing on patients with CD who underwent surgery with or without drug therapy with biologics. Patient data from these studies were extracted and compared depending on the intervention.

All eligible studies were reviewed and selected according to the following primary endpoints: Clinical recurrence and surgical recurrence (= need for re-intervention/dilatation). Clinical relapse was defined based on Crohn’s Disease Activity Index (CDAI) scores [5]. The introduction or intensification of CD-related medications is also defined as clinical recurrence. Surgical recurrence was defined as the need for re-operation in the ileocecal resection arm and the need for surgery in the biologics arm. The studies were assigned to one of the three treatment groups (ileocecal resection, use of biologics (infliximab or adalimumab), resection with following use of biologics) in an Excel table.

### Data items

From each trial, data were extracted regarding study design, publication year, type of therapy, application info, follow-up times, number of participants, gender, recurrence-free years, target criteria (clinical, endoscopic, or surgical recurrence) and their time of onset, pre- and post-medication and pre-operations.

### Synthesis methods

A consort diagram was prepared for a more straightforward presentation of study identification throughout the inclusion or exclusion of studies. Because not all variables from the original Excel file were needed for our meta-analysis, a table was created for each treatment option (ileocecal resection, biologics and combination therapy). The tables contained the variables study number, author, year, participants, male, female, follow-up time and definition of relapse.

### Statistical analysis

Because there are almost no trials that describe a direct head-to-head comparison of medical and surgical treatment regimens, we performed a meta-analysis of proportions (incidence). The incidence represented the ratio of patients with complications to the total number of patients in that study at a specific time. This analysis was performed at various times (e.g. 12 and 60 months). To perform a combined analysis, we applied a random intercept logistic regression model with covariate treatment (surgery, biologics and combination) and time because several studies reported more than one time that induced correlated data. We used the model-based odds ratio with a 95% confidence interval (*CI*) to examine treatment comparisons and time effects.

Analysis of heterogeneity of incidences across studies was initially performed using Cochran’s *Q*-test. The degree of heterogeneity was also quantified using *I*^2^ values. The *I*^2^ statistic describes the percentage of variation across studies due to heterogeneity rather than chance. This meta-analysis estimated the heterogeneity variance tau^2^ based on the maximum likelihood estimate according to the random effects logistic regression model.

Publication bias was investigated using Egger’s regression test and represented by funnel plots for surgical and clinical recurrence [6] (Suppl Figs. 1 and 2).

All statistical analyses were performed using R (version 1.4.1717) and the corresponding packages lme4 [7] and metaprop [8].

## Results

**Fig. 1:**
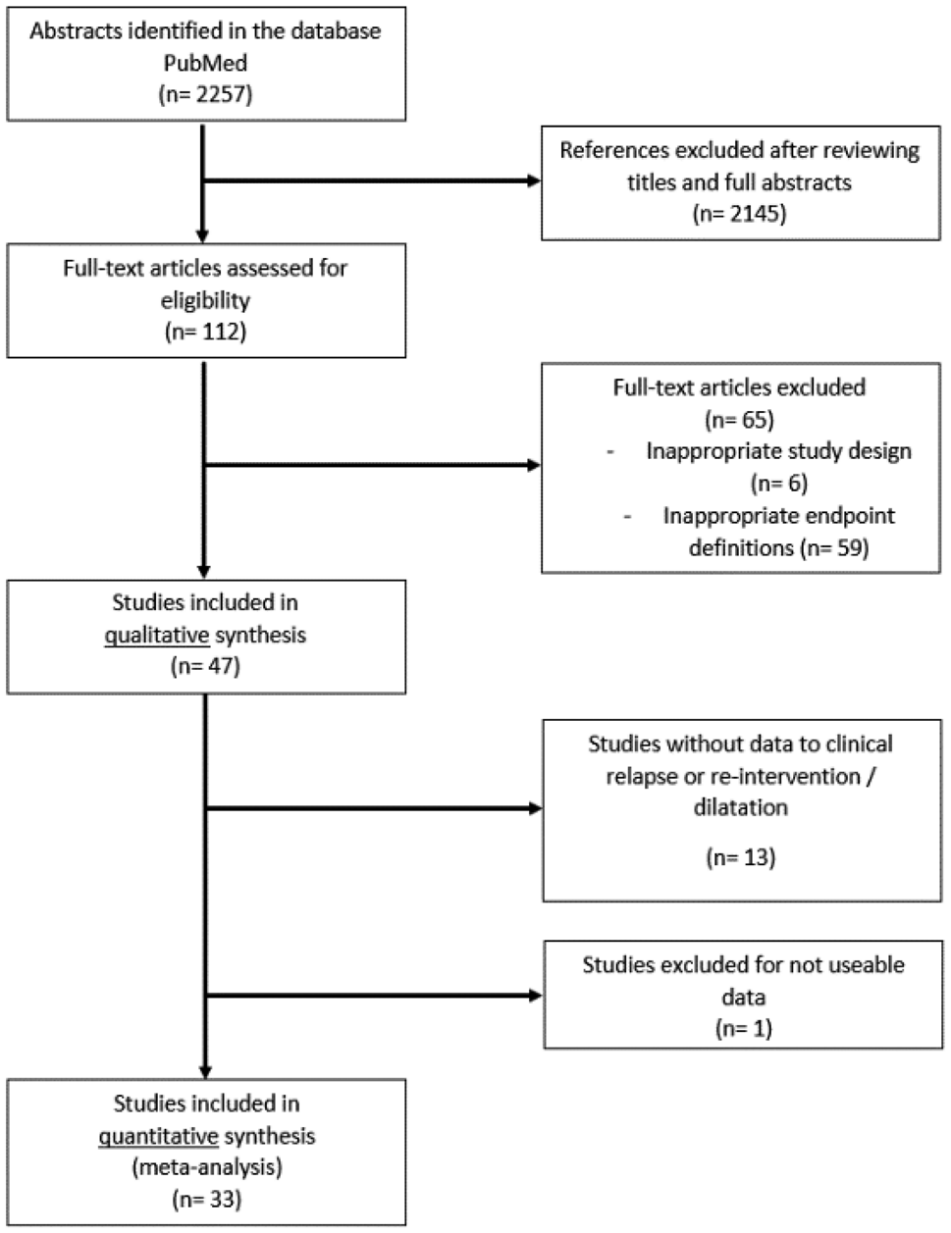
Consort diagram showing number of studies evaluated for inclusion in this meta-analysis and the reasons for exclusion of studies

Two authors (SK, AS) performed a literature search in PubMed to locate studies evaluating the efficacy of biologics, surgical resections and combination therapies (resection and post-operative introduction of biological therapy). Our search strategy identified 2257 references (Fig. 1).

Of these, 112 were eligible, published between 1983 and 2021. Sixty-five were excluded because of inappropriate study designs (follow-up only for 30 days) or other endpoint definitions for achieving recurrence. One article was excluded because of inappropriate data sets. After reviewing all studies and collecting the results, we determined that the endpoint of endoscopic recurrence was not suitable for comparing different relapse-preventing therapies because the possible treatment goals of the interventions differed across studies. While surgery resects the affected bowel segment and optimally removes all endoscopic lesions, the administration of biologics can lead to mucosal healing. Still, it may (in many patients) only prevent the endoscopic lesions from progressing and spreading. Therefore, the data collected on endoscopic recurrence were not included. This exclusion affected five studies in the ileocecal resection arm, two in the biologics arm, and six in the combination arm. Overall, 33 studies with 4220 participants were considered in our meta-analysis. For surgical recurrence, we found publication bias according to Egger’s regression test with a bias equal to —2.6518 and *p*-value = 0.0143 (Supplementary Fig. 1). For clinical recurrence, there was no publication bias (bias — 0.4064 and *p*-value = 0.6878) (Supplementary Fig. 2).

Most studies in our meta-analysis were published in the early 2000s. Of the 33 studies, ten described patients with ileocecal resections as one of the three treatment groups; 12 focused on medical treatment with infliximab or adalimumab; and the remaining 11 included patients with sequential combination therapies with surgical resections and subsequent use of infliximab or adalimumab. Baseline information and patient characteristics are shown in Tables 1–3.

**Table 1:** Characteristics of the included ileocecal resection studies

**Reference**	**Author**	**Year**	**Participants**	**Male**	**Female**	**Follow-up**	**Definition of relapse**
[37]	Aaltonen et al.	2018	92	55	37	60 mo	Need for endoscopic dilatation or surgical reintervention
[33]	Kim et al.	1997	181	79	102	60 mo	Re-OP
[38]	Rink et al.	2014	119	37	82	60/120 mo	Symptomatic restenosis with need for dilatation or Re-OP
[9]	De Buck van Overstraeten et al.	2017	538	215	323	12/60/120 mo	Need for medication or intensification of treatment; Re-OP
[34]	Yamamoto et al.	1999	141	17	45	96 mo	Re-OP
[10]	Margagnoni et al.	2011	212	120	92	120 mo	Symptoms with need for steroids or budesonid in the presence of endoscopic + / − radilogical recurrence; Re-OP
[12]	Cullen et al.	2007	139	55	84	60/84/120 mo	Symptoms + radilogical/endoscopical proof; Re-OP
[35]	Rutgeerts et al.	1990	89			12/36/60/96 mo	Rutgeerts´ ≥ i1; Re-OP
[11]	Rivière et al.	2021	365	155	210	6.2/88 mo	Rutgeerts´ ≥ i1; Dilatation or Re-OP; need for IMM or biologicals
[36]	Riss et al.	2014	116	66	50	60/120 mo	Re-OP

Table 1 shows the characteristics of the studies included in the meta-analysis for the ileocecal resection treatment. Noticed that most of the studies defined relapse as the need for re-operation (Re-OP). The studies in the table are broken down by author, publication year, number of participants, gender, follow-up in months, and definition of relapse

**Table 2:** Characteristics of the included biologics studies

**Reference**	**Author**	**Year**	**Participants**	**Male**	**Female**	**Follow-up**	**Drug**	**Definition of relapse**
[13]	Colombel et al.	2010	169	84	85	6 mo	Infliximab	Need for corticosteroids; continuation of ulcerations
[14]	Schnitzler et al.	2009	614	240	374	55.3 mo	Infliximab	Major abdominal surgery (MAS); need for intervention (clinical)
[2]	Stevens et al.	2020	65	19	46	17/63.5 mo	Infliximab	Surgery; need for further IFX or switch of Medication
[39]	Feagan et al.	2008	517	197	320	12.8 mo	Adalimumab	Major abdominal surgery (MAS)
[15]	Ho et al.	2008	22	8	14	12 mo	Adalimumab	Surgery; ADA dose escalation
[16]	Macaluso et al.	2019	214	118	96	2.7/12 mo	Infliximab, Adalimumab	Need for steroids + symptoms
[17]	Kestens et al.	2013	100	45	55	12/ 24 mo	Infliximab, Adalimumab	Surgery; need for steroids
[21]	Hinojosa et al.	2007	36	11	25	1 mo	Adalimumab	CDAI ≥ 150
[22]	Seiderer et al.	2007	16	8	8	1.8/5.5 mo	Adalimumab	CDAI ≥ 150
[18]	Colombel et al.	2015	62			6 mo	Infliximab	CDAI ≥ 150; need for steroids; continuation of ulcerations
[19]	Peyrin-Biroulet et al.	2007	24	5	19	1/12 mo	Adalimumab	CDAI ≥ 150; need for steroids
[20]	Cordero-Ruiz et al.	2011	25	10	15	5.5/11 mo	Adalimumab	CDAI ≥ 150; need for steroids

Table 2 shows the characteristics of the studies included in the meta-analysis for the treatment with biologics. Noticed that most of the studies defined relapse as the need for steroids and by using the CDAI-score. The studies in the table are broken down by author, publication year, number of participants, gender, follow-up in months, which drug was given (IFX and/or ADA), and definition of relapse

**Table 3:** Characteristics of the included combination therapy (resection and biologics) studies

**Reference**	**Author**	**Year**	**Participants**	**Male**	**Female**	**Follow-up**	**Intervention**	**Definition of relapse**
[23]	Yoshida et al.	2012	15	11	4	12/36 Mo	OP + IFX/placebo	Rutgeerts´ ≥ i2; CDAI > 150; Re-OP
[24]	Fukushima et al.	2018	19	17	2	12/24 Mo	OP + IFX/placebo	Rutgeerts´ ≥ i3; CDAI > 150
[25]	Regueiro et al.	2009	11	6	5	12 mo	OP + IFX/placebo	Rutgeerts´ ≥ i2; CDAI > 150; Re-OP
[30]	Aguas et al.	2012	29	16	13	12 mo	OP + ADA	Rutgeerts´ ≥ i2; symptoms + need for medication change/ Re-OP
[26]	Asada et al.	2018	26	19	7	12/24 mo	OP + ADA	Rutgeerts´ ≥ i2; CDAI > 150; Re-OP
[31]	Cañete et al.	2019	152	85	67	18 moo	OP + ADA/ IFX	Rutgeerts´ ≥ i3; symptoms + disease activity (ileocolonoscopy or MRE)
[27]	Marteau et al.	2006	50	21	29	6 mo	OP + placebo	Rutgeerts´ ≥ i2 + colonic lesions; CDAI > = 200
[28]	Rutgeerts et al.	2005	40	20	20	3/12/24/36 mo	OP + Placebo	endoscopic recurrence score ≥ i2; symptoms + CDAI > 250
[32]	Hanauer et al.	2004	40	18	22	24 mo	OP + placebo	Rutgeerts´ ≥ i2; clinical recurrence grading scale > = i2
[41]	Araki et al.	2014	100	74	26	36/51 mo	OP + IFX/ placebo	Re-OP
[29]	Mowat et al.	2016	112	45	67	36 mo	OP + placebo	Rutgeerts´ ≥ i2; CDAI > 150 + 100Pkt-Anstieg

Table 3 shows the characteristics of the studies included in the meta-analysis for the combination treatment with resection and biologics. Noticed that most of the studies defined relapse by using Rutgeerts’ score and CDAI-score. The studies in the table are broken down by author, publication year, number of participants, gender, follow-up in months, type of intervention, and definition of relapse

### Clinical recurrence

Clinical recurrence after ileocecal resection was defined as symptoms and the need for new medications like systemic steroids, immunosuppressives and biologics [9–11] or symptoms and radiological/endoscopic proof of recurrence [10, 12]. In the biological group, clinical recurrence was defined as the need for steroids or other medical intervention (dose intensification, switch to other biologics or introduction of combination therapies) [2, 13–20]. Other studies defined clinical recurrence based on a CDAI score ≥ 150 [18–22]. In the combination group (surgery + biologics), the CDAI score was used to define clinical recurrence. In most studies in the combination group, CDAI > 150 defined recurrence of disease [23–26], whereas three other studies [27–29] set their limit on a higher CDAI score or the need for an additional criterion (Table 3). In two studies, the patients had to show symptoms and a need for intervention (medication, re-operation) or radiologically confirmed disease activity [30, 31]. In one study, the authors used their clinical recurrence scale and defined recurrence as a Rutgeerts score ≥ i2 [32].

**Table 4:** Odds ratio (*OR*) with corresponding 95% confidence interval (*CI*) for clinical and surgical recurrence with combination therapy or biologics in relation to recurrence rates with ileocecal resection. The *OR* for the follow-up shows an increase in surgical and clinical recurrence rates over the years for all three treatment options

**Characteristic**	**Clinical recurrence**		**Surgical recurrence**	
	*OR* (95% *CI*)^1^	*p*-value	*OR* (95% *CI*)1	*p*-value
Therapy				
Surgery	–		–	
Biologics	2.50 (1.53 to 4.08)	< 0.001	3.60 (1.06 to 12.3)	0.041
Both	0.76 (0.40 to 1.42)	0.39	0.33 (0.04 to 2.69)	0.30
Follow-up (year)	1.19 (1.16 to 1.23)	< 0.001	1.31 (1.25 to 1.38)	< 0.001
Tau^2	0.13		1.17	

^1^*OR* odds ratio, *CI* = confidence interval, *Tau^2* heterogeneity variance

### Surgical recurrence

In the ileocecal resection group, surgical recurrence was defined by the need for re-operation [9, 10, 12, 33–36] or (in some studies) endoscopic dilatation [11, 37, 38]. In the biological group [2, 15, 17], the need for re-intervention/dilatation was already considered a surgical recurrence. In two studies [14, 39], only the need for major abdominal surgery was rated as a recurrence.

### Overall evaluation

**Fig. 2:**
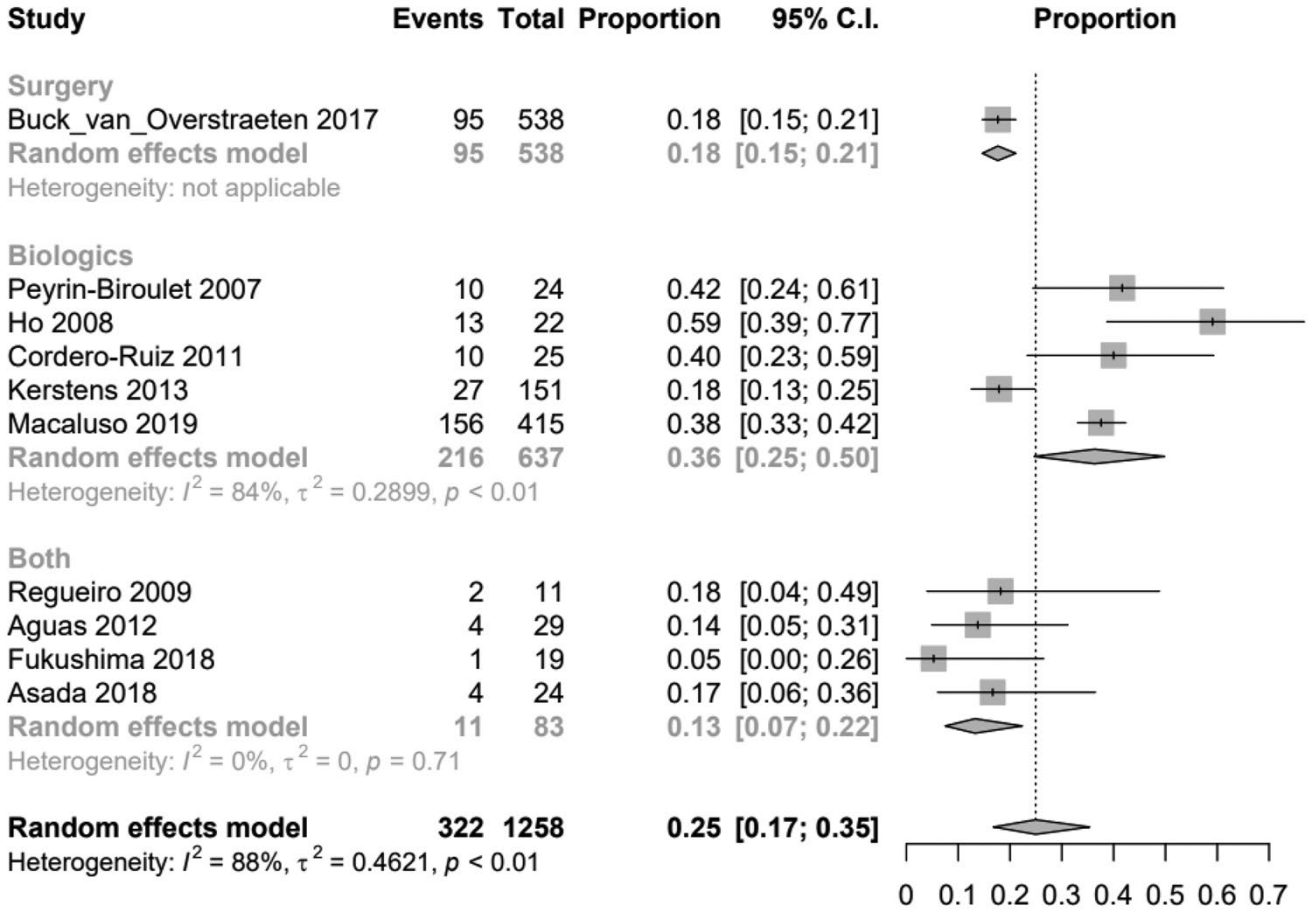
Forest plot comparing clinical recurrence after 12 months in patients with ileocecal resection (surgery), therapy with biologics or combination therapy (both)

**Fig. 3:**
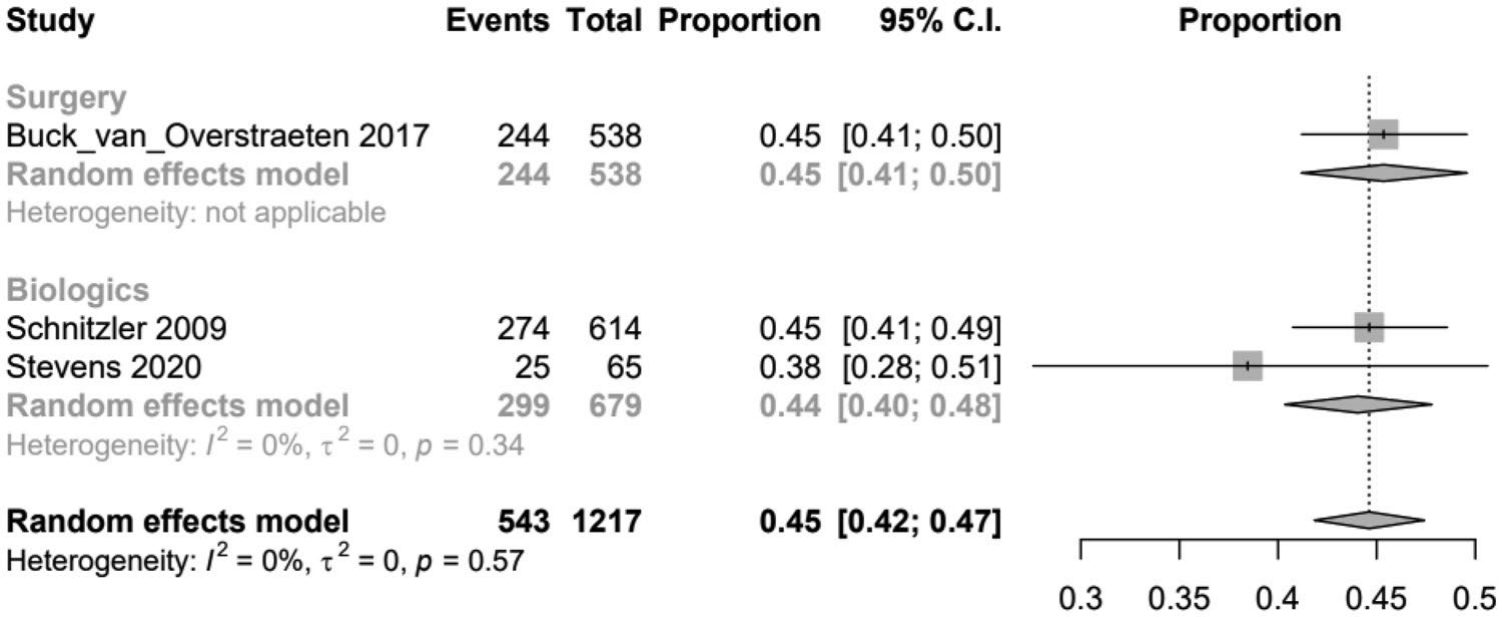
Forest plot comparing clinical recurrence after 60 months in patients with ileocecal resection (surgery) or therapy with biologics

**Fig. 4:**
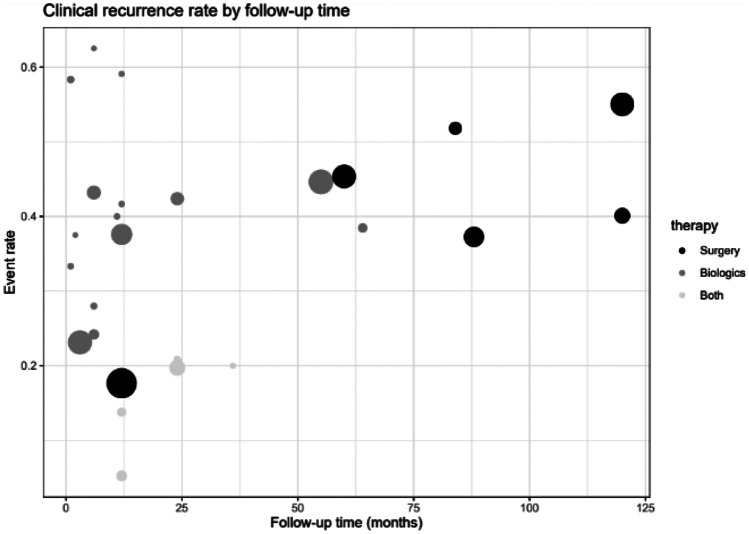
Bubble plot for clinical relapse by follow-up time. Size of the circles corresponds to study size. Over time, probability of a clinical relapse increases for all three therapy options

**Fig. 5:**
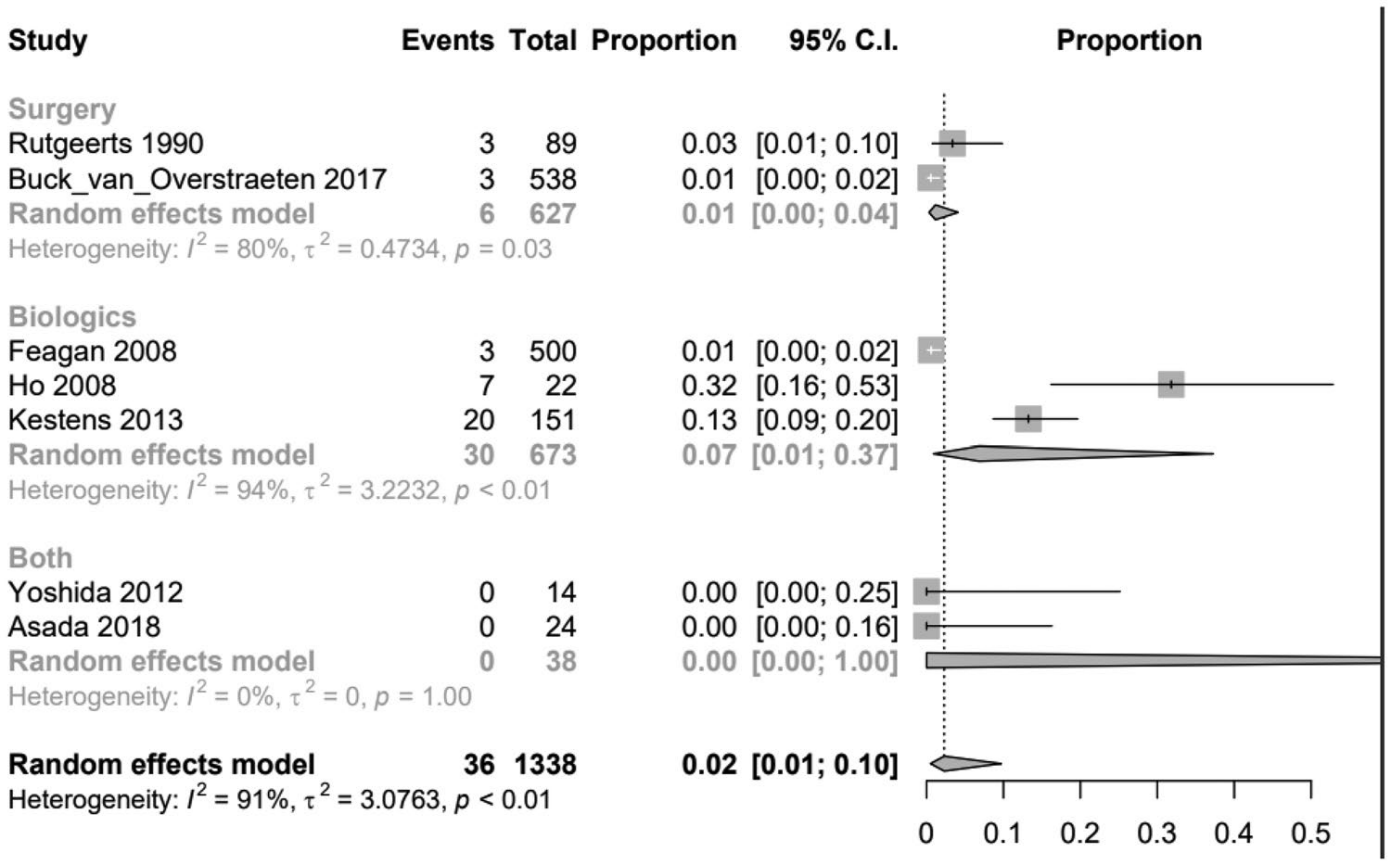
Forest plot comparing surgical recurrence after 12 months in patients with ileocecal resection (surgery), therapy with biologics or combination therapy (both)

**Fig. 6:**
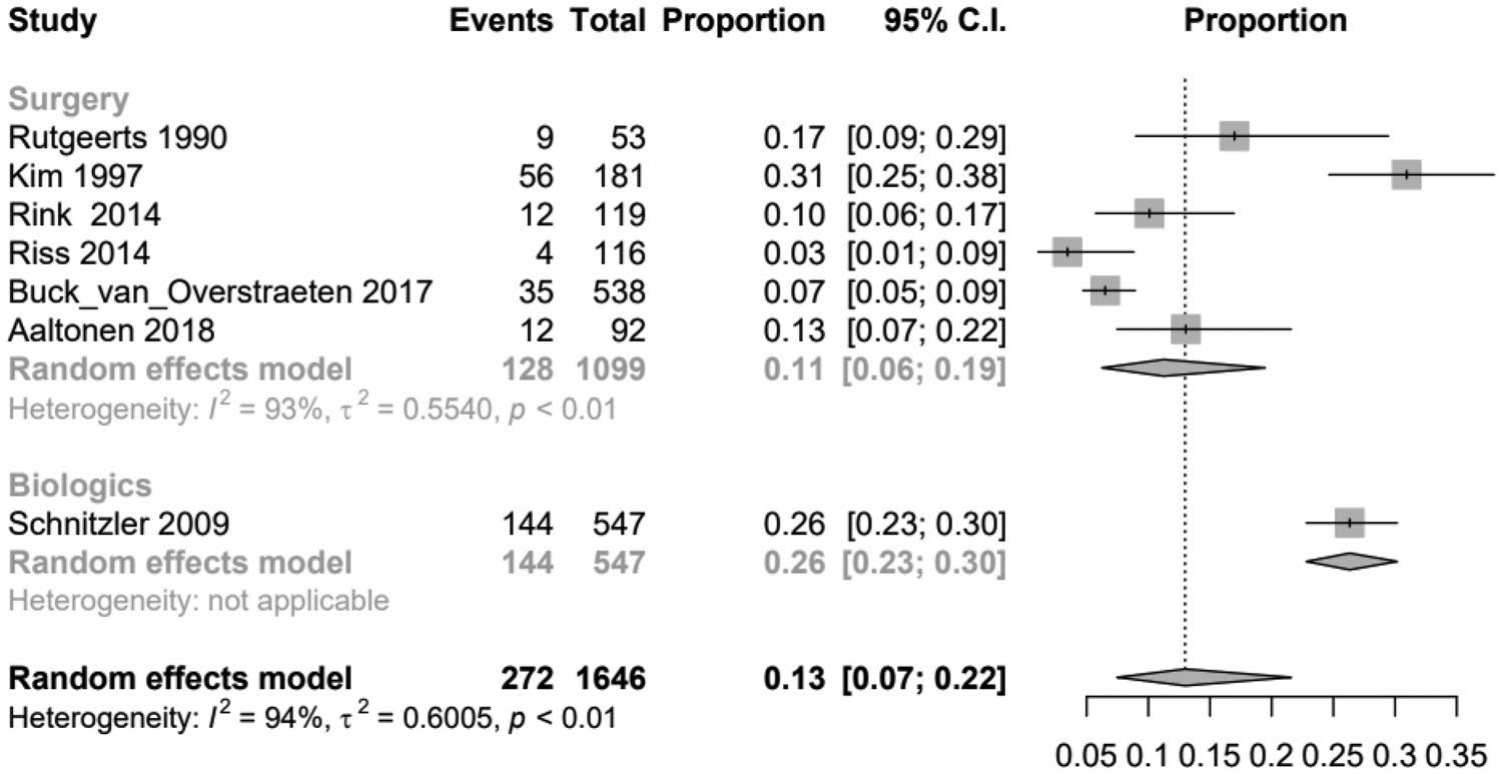
Forest plot comparing surgical recurrence after 60 months in patients with ileocecal resection (surgery) or therapy with biologics

**Fig. 7:**
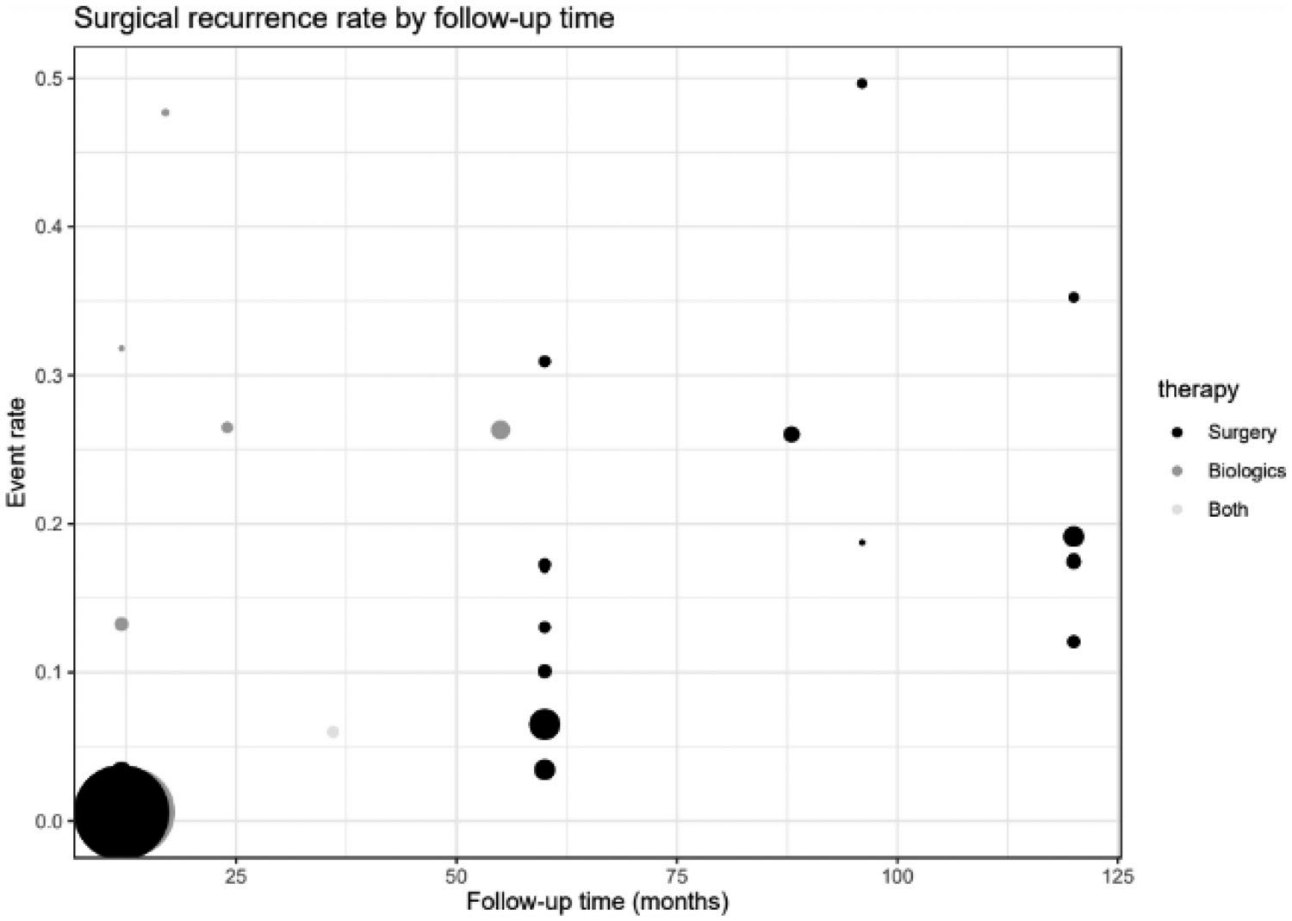
Bubble plot for surgical relapse by follow-up time. Size of the circles corresponds to study size. Over time, probability of a surgical relapse increases for all three therapy options

To create the opportunity to compare the three therapy options, ileocecal resection, use of biologics or combination of surgery and biologics regarding the different follow-ups, a random effects logistic regression model was applied for clinical and surgical recurrences. The following graphs (Figs. 2, 3, 5 and 6) show the probabilities of a clinical or surgical recurrence for the various therapy options after 12 or 60 months using a scale. The number 0 means no recurrence occurred in any study participant, and (for example) the value 0.5 means that half the participants suffered a recurrence within the defined period. A bubble plot for clinical and surgical recurrences was generated to clarify the results and compare the various therapy options regarding their recurrence rates (see below). These plots represent the logistically calculated recurrence rates of the three treatment options (surgery, biologics and both) over time. The circles in the plot represent the individual studies. Larger circles represent larger study populations. The *y*-axis shows the event rate (i.e. clinical recurrence (Fig. 4) and surgical recurrence (Fig. 7). The follow-up time in months is plotted on the *x*-axis.

After 12 months, the lowest risk of clinical recurrence was observed in the combination group, 0.13 (95% *CI*, 0.07–0.22), followed by the surgical group at 0.18 (95% *CI*, 0.15–0.21) and the biologic group at 0.36 (95% *CI*, 0.25–0.50). The results had to be checked for significance to interpret the plot below. In the plot on clinical recurrence, the combination therapy ‘both’ also appeared as the best therapy option with the lowest probability of recurrence. However, this assumption was not statistically significant, as the *p*-value (Pr) for the combination therapy was 0.71. This finding suggests that ileocecal resection is associated with the best outcome in terms of clinical recurrence after 12 months (Fig. 2).

After 60 months, the clinical recurrence rates were similar between the surgery and biologics groups, with 0.45 (95% *CI*, 0.41–0.50) in the surgery group and 0.44 (95% *CI*, 0.40–0.48) in the biologics group. No studies were found for 60 months from the therapy regime ‘both’. However, the results of the biologics group were not statistically significant, as their *p*-value (Pr) was 0.34 (Fig. 3).

In the first 25 months, clinical relapses occurred primarily with biologics therapy (event rate 0.2–0.6). Over time, recurrence rates increase with surgical treatment (event rate 0.35–0.55) (Fig. 4). These findings suggest that patients in the three therapy groups suffer a higher risk of recurrence over time.

After 12 months, the lowest risk of surgical recurrence was observed in the combination group (0.00 [95% *CI*, 0.00–1.00]), followed by the surgical group (0.01 [95% *CI*, 0.00–0.04]) and the biologic group (0.07 [95% *CI*, 0.01–0.37]). The combination group ‘both’ results were insignificant and therefore not meaningful, as their *p*-value (Pr) was 1.00 (Fig. 5).

After 60 months, the surgical recurrence rates were 0.11 (95% *CI*, 0.06–0.19) in the surgery group and 0.26 (95% *CI*, 0.23–0.30) in the biologics group. There were no studies from the combination therapy group for the probability of recurrence after 60 months. The results of the two types of therapy, surgery and biologics, were statistically significant (Fig. 6); more than twice as many surgical recurrences occurred in the biologics group after 60 months as in the surgical group.

In the first 25 months, surgical relapses occurred primarily with biologics therapy (event rate between 0.1 and 0.5). From 60 months and beyond, the recurrence rates increased with surgical treatment. Just as in the bubble plot for clinical recurrence, this plot showed an increase in surgical recurrence rates in all therapy groups over time (Fig. 7).

Table 4 displays the OR for clinical and surgical recurrence with combination therapy or biologics concerning recurrence rates with ileocecal resection. The OR for the biologics group was 2.50 (95% *CI* 1.53–4.08, *p*-value < 0.001), suggesting a higher risk of clinical recurrence with biologics than with ileocecal resection.

The table also shows that the risk of clinical recurrence increased by 1.19 with every year of therapy, regardless of therapy type. The OR for the biologics group was 3.60 (95% CI 1.06–12.3, p-value 0.041), suggesting that the surgical recurrence risk was higher than under surgical therapy. Here, the surgical recurrence risk also increased by 1.31 per year regardless of therapy type. The clinical and surgical recurrence rates under combination therapy ‘both’ were not statistically significant.

## Discussion

Because CD is a chronic inflammatory condition that progresses and cannot be cured, it is critical to achieving sustained clinical remission. Current valid guidelines recommend checking the indications for surgery before starting immunosuppressive therapy (e.g. with biologics). Evidence for this recommendation was provided by the LIR!C trial using a head-to-head comparison between biologic administration and surgery with limited and predominantly inflammatory terminal ileitis for whom conventional treatment was unsuccessful [2]. This ground-breaking study was neither confirmed nor refuted. Against this background, we performed a detailed meta-analysis comparing surgical and drug treatment regarding the likelihood of recurrence.

In summary, surgical resection showed the best results over time in surgical and clinical recurrence, with the lowest recurrence rates compared to treatment with biologicals (Table 4). Most included studies only considered one intervention and its outcome and not the individual treatment options in terms of different endpoints. Therefore, our meta-analysis acquired sufficient data from ileocecal resection, biologics (infliximab, adalimumab) and a combination of surgery and biologics. Based on a logistic regression model, 33 studies were compared regarding clinical recurrence and repeat surgical interventions. The finding might explain the robustness of the outcomes that the probability of recurrence increased for all three treatment options over time (Figs. 4 and 7, Table 4). The endpoint of endoscopic recurrence (initially considered) was not included in interpreting the results. However, endoscopic findings are becoming increasingly crucial in therapy management and will be indispensable for optimising and individualising therapy in the future. Orlando et al. showed that it is helpful to perform early endoscopic follow-up to detect recurrences in time and counteract them [40].

A limitation of our study was that the studies set their follow-ups at different, non-uniform time points. Therefore, we used a logistic regression model to compare them. Nevertheless, it would have been desirable for the long-term course if several follow-ups had been made and reported in each study. Furthermore, we did not set a time frame for the publication year of the studies and included older studies from the 1990s (Tables 1–3). It must be kept in mind that some newer surgical procedures and biologics have only been developed recently. Moreover, the studies often did not have identical definitions of when relapse was reached (e.g. in the case of need for re-intervention/dilatation). Some studies defined minor interventions such as endoscopic dilatations as recurrence. Other studies defined only major surgeries as recurrence (Tables 1–3). It must also be kept in mind that different study-specific threshold values were set when using the CDAI for clinical recurrence and that this score also depends on the subjective perception of the patient (Tables 2 and 3). Furthermore, it was not able to differentiate between inflammatory or stenotic condition as an indication for re-intervention in the prior surgery group.

Our endpoint of endoscopic recurrence (defined initially) was not included in our interpretation because the two interventions, ileocecal resection and use of biologics, had different therapeutic starting points concerning endoscopy. Ileocecal resection aims to remove affected bowel segments with inflammatory and endoscopic abnormalities. This is considered an endoscopic recurrence if endoscopic abnormalities reappear post-operatively in the sections that were not resected. On the other hand, biologics attempt to treat existing endoscopic ulcerations/abnormalities. In this case, endoscopic recurrence cannot be used as the endpoint, as the affected foci were never thoroughly removed and subsequently recurred. The only statement that can be made concerns how biologics affect mucosal healing. Statements were only made about the biologics infliximab and adalimumab, as there were too few data for others (e.g. ustekinumab and vedolizumab). Finally, we could not achieve a homogeneous patient population with the same baseline characteristics because the individual studies each set different inclusion and exclusion criteria. Therefore, the patients differed in prior medication and surgery, concomitant medication, sex, smoking status and age.

Our meta-analysis is further limited by publication bias in the surgical recurrence arm. This bias could not be mitigated. An additional literature search of PubMed and the internet yielded no further matching manuscripts.

In summary, our meta-analysis suggests that surgical resection is associated with better outcomes in terms of clinical recurrence and the need for re-intervention/dilatation than monotherapy with biologics or combination therapy. The results are robust and well suited for counselling patients before starting immunomodulatory treatment. They also support the recommendations in the current guidelines for treating CD [2].

## Supplementary Information

Below is the link to the electronic supplementary material.

Supplementary file1 (DOCX 56 KB)
