# Correction to: Disease recurrence in patients with Crohn’s disease after biologic therapy or surgery: a meta-analysis

**DOI:** 10.1007/s00384-022-04277-6

**Published:** 2022-11-05

**Authors:** Sarah Kneißl, Johannes Stallhofer, Peter Schlattmann, Andreas Stallmach

**Affiliations:** 1 Department of Internal Medicine IV (Gastroenterology, Hepatology, and Infectious Diseases), Jena University Hospital, Am Klinikum 1, 07747 Jena, Germany; 2 Institute for Medical Statistics, Informatics and Data Science, University Hospital Jena, Bachstr. 18, 07743 Jena, Germany

**Correction to: International Journal of Colorectal Disease (2022) 37:2185-2195** https://doi.org/10.1007/s00384-022-04254-z

The original published version of the above article contained errors. The authors’ request that the following changes be noted **[**Bold text used to highlight corrected area**]**.

1. The data in the sections under Abstract and Results has been updated.
**Abstract**
  **Results The need for re-intervention with medication or surgery due to surgical or clinical recurrence increased over time.** The recurrence rates in patients after ileocecal resection were lower than the rates under biologic therapy. The odds ratio for clinical recurrence under biologics versus after surgical treatment was 2.50 (95% confidence interval [CI] 1.53–4.08, p-value < 0.001). The odds ratio for surgical recurrence under biologics versus after surgery was 3.60 (95% CI 1.06–12.3, p-value 0.041).
**Results**
**Overall evaluation**
  **After 60 months, the surgical recurrence rates were 0.11 (95%** ***CI*, 0.06–0.19) in the surgery group and 0.26 (95% *CI*, 0.23–0.30) in the biologics group**. There were no studies from the combination therapy group for the probability of recurrence after 60 months. The results of the two types of therapy, surgery and biologics, were statistically significant (Fig. 6); more than twice as many surgical recurrences occurred in the biologics group after 60 months as in the surgical group.
2. Reference, Table and Figure citations of the sections ‘**Statistical analysis, Clinical recurrence, Surgical recurrence, Overall evaluation and Discussion’** has been rearranged and corrected.
3. Figure 1 caption has been corrected to “**Fig. 1** Consort diagram showing number of studies evaluated for inclusion in this meta-analysis and the reasons for exclusion of studies”
4. In Table 4, the data entry under the ‘Clinical recurrence’ column with the Characteristic ‘Biologics’ should have been **2.50** instead of **250.**
5. The figure caption for **Figure S2** has been corrected. The Supplementary material was replaced by the new material.

The original article has been corrected.

