# Supplementary material for: Disease recurrence in patients with Crohn’s disease after biologic therapy or surgery: a meta-analysis
Source: Int J Colorectal Dis. 2022 Sep 23;37(10):2185–95. doi: 10.1007/s00384-022-04254-z (PMC9560971; doi:10.1007/s00384-022-04254-z)
Supplement: Supplementary file 1 — Supplementary file1 (DOCX 56 KB) [file 384_2022_4254_MOESM1_ESM.docx]

**Supplement:**


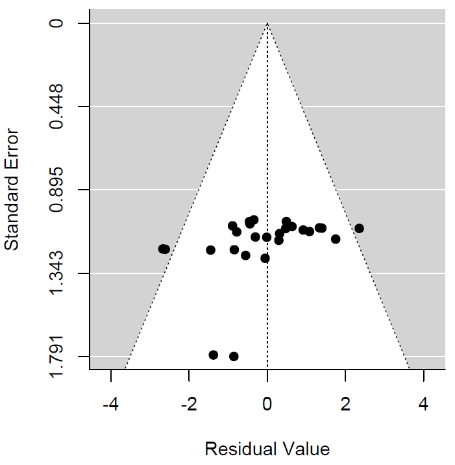


Figure 1: Publication bias represented by funnel plot for surgical recurrence. The standard error (y-axis) provides a measure of the precision of the residual value (x-axis) as an estimate of the population parameter. Therefore, study size increases the precision of the estimated effect and decreases the size of the standard error. Inhomogeneous distribution of studies (points) within the funnel indicates a publications bias.


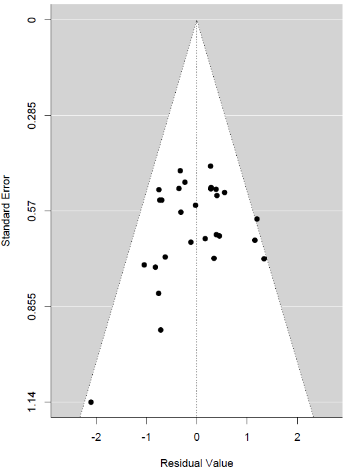


Figure 2: Publication bias represented by funnel plot for clinical recurrence. The standard error (y-axis) provides a measure of the precision of the residual value (x-axis) as an estimate of the population parameter. Therefore, study size increases the precision of the estimated effect and decreases the size of the standard error. Homogeneous distribution of studies (points) within the funnel makes publication bias improbable.
